# Chemically Grafting Carbon Nanotubes onto Carbon Fibers for Enhancing Interfacial Properties of Fiber Metal Laminate

**DOI:** 10.3390/ma13173813

**Published:** 2020-08-28

**Authors:** Fan Ji, Cheng Liu, Yubing Hu, Shengnan Xu, Yiyan He, Jin Zhou, Yanan Zhang

**Affiliations:** 1 College of Materials Science and Engineering, Nanjing Tech University, Nanjing 211816, China; 201861203069@njtech.edu.cn (F.J.); 201861203146@njtech.edu.cn (S.X.); 201961203128@njtech.edu.cn (Y.H.); 2 Institute for Composites Science Innovation (InCSI), School of Materials Science and Engineering, Zhejiang University, Hangzhou 310027, China; 11926075@zju.edu.cn; 3 National Special Superfine Powder Engineering Research Center of China, Nanjing University of Science and Technology, Nanjing 210014, China; hyb@njust.edu.cn; 4 Key Laboratory of Special Energy Materials, Nanjing University of Science and Technology, Ministry of Education, Nanjing 210094, China; 5 School of Mechanical Engineering, Xi’an Jiaotong University, Xi’an 710049, China; jin.zhou@xjtu.edu.cn

**Keywords:** chemical grafting, carbon fiber, carbon nanotube, fiber metal laminates

## Abstract

This paper primarily investigates the effects of chemically grafted modified carbon fibers on the bonding properties of fiber metal laminates (FMLs). Relative elemental content on the carbon fibers’ surface was performed via X-ray photoelectron spectroscopy (XPS). Scanning electron microscopy (SEM) was utilized to observe the material microstructure. The effect of chemically grafted carbon fibers on the bond strengths of FMLs was experimentally investigated through lap joint testing. The carbon nanotubes (CNTs) grafting concentration and curing conditions of the samples were also investigated. The test results demonstrate that grafting concentrations of 0.1, 0.2, and 0.3 mg/mL CNT solution increased the bond strength of the cured samples under vacuum conditions by 63.51%, 87.16%, and 71.56%, respectively. In addition, the bond strengths of vacuum-cured samples were also increased.

## 1. Introduction

Fiber metal laminates (FMLs) are hybrid composites of alternating layers of high-strength fiber composites and alloys, cured under pressure and temperature. FML structures combine the performance advantages of fiber composites with metal materials to overcome the performance defects of each material separately [1,2,3]. They possess a series of excellent properties, such as high specific modulus, high specific strength, and heat and corrosion resistance, and have been widely used in transportation, sporting goods, and aerospace applications [4,5]. The performance of FMLs is heavily dependent on the strength of the interface between the fiber-reinforced composites and metal sheets [6]. So far, many studies have concentrated on metal layer surface treatment technologies such as acid etching [7,8], sandblasting [9,10], and anodization [11,12]. Additionally, there are numerous reports on resin modification by adding nano-reinforcements to improve the composite performance [13,14].

Carbon fiber has excellent tensile strength, high modulus, high stiffness, light weight, and great thermal resistance, making carbon fiber composite materials ideal structural materials [15]. The next generation of FMLs consists of carbon fiber composites and titanium alloy sheets. However, untreated carbon fibers’ surfaces are smooth and chemically inert, which result in poor matrix compatibility [16]. There are plenty of ways to implement carbon fiber modification, including sizing [17,18], electrophoretic deposition [19,20], high-energy irradiation [20,21], and chemical grafting [22,23,24]. At present, a large number of studies have revealed that the performance of fiber composites can be improved with carbon fiber modification. For example, Peng et al. reported that chemically functionalized carbon fiber with poly (amido amine) provided an 85% increase in the interlaminar shear strength (ILSS) of carbon fiber composites [25]. Wu et al. showed an increase of 53.27% in ILSS and 40.92% in interfacial shear strength (IFSS) compared with those of untreated composites through direct grafting of 3-aminopropyltriethoxysilane (APS)-functionalized silica nanoparticles (SiO_2_-APS) onto the carbon fiber surface [26]. Zhao et al. attached carbon nanotubes (CNTs) to carbon fibers using polyhedral oligomeric silsesquioxane (POSS), and IFSS increased by 105% [27]. Therefore, it is pertinent to study the interfacial properties of modified carbon fiber-reinforced FMLs. The interfacial performance has an important influence on the overall mechanical properties of the FMLs. For instance, Faggiani [28] used an in-layer damage model to simulate the failure mechanism of carbon fiber composite materials, including shear failure, tensile fracture and fiber damage. It shows that the toughness and shear strength of the composite laminate is poor, and the material is prone to delamination and ultimate failure. To enhance laminate performance, scientists have conducted a lot of research on the interface properties of composite laminates. Currently, the main research object is the metal/resin interfacial with relatively weak performance. Few studies have been performed on the interface between fiber and resin and its impact on laminate performance. In view of this, this paper focuses on the effects of chemically grafting CNTs on the surface of carbon fibers with respect to the bonding properties of composite laminates.

Chemical grafting is a flexible and convenient method to modify carbon fiber, and it can obtain controlled and active structures on the surface of carbon fibers [29]. Owing to these advantages, the chemical grafting method is employed in this work. In the present research, carboxylic acid-functionalized multi walled carbon nanotubes (MWCNTs) were selected as nano-reinforcing materials due to their excellent properties [30]. The purpose of using MWCNTs is to enhance mechanical bonding between the fiber and resin using van der Waals interactions, thereby improving the interfacial performance of the FMLs [31]. Furthermore, melamine can be used as a coupling agent due to its economic value and higher amino group density. Under the action of a condensing agent, the amino group provided by melamine reacted with the carboxyl group on the oxidized carbon fiber and carboxylic acid-functionalized CNT surface, respectively. In this experiment, the concentrations of various CNT solutions and the of the samples relative to the bonding strength of the FMLs were invented.

## 2. Experimental

### 2.1. Materials

Carbon fiber weaves (T-300 12k) were purchased from Toray Co., Tokyo, Japan. Carboxylated multi-walled carbon nanotubes (diameter 50 nm, length 1–2 μm) were received from Nanjing Xianfeng Nano Material Technology Co., Ltd., Nanjing, China. Melamine (Shanghai Linen Science and Technology Development Co., Ltd., Nanjing, China) was used as the coupling agent. 2-(7-Azabenzotriazol-1-yl)-N, N, N’, N’-tetramethyluronium hexafluorophosphate (HATU) was supplied by Sinopharm Chemical Reagent Co., Ltd., Nanjing, China. Titanium alloy sheet (TA2) was obtained from Baoji Titanium Industry Co., Ltd., Baoji, China. All other chemicals were procured from Nanjing Chemical Reagent Co., Ltd., Nanjing, China. In this experiment, YT-CC302Q low-viscosity quick-drying epoxy resin provided by Huibai New Material Technology Co., Ltd., Shanghai, China, was used. The epoxy resin and curing agent were formulated according to the weight ratio 3:1.

### 2.2. Fabrication of Oxidized Carbon Fiber Weaves

A layer of carbon fiber weaves was placed into acetone/ethanol (1/1, *v*/*v*) solution at 50 °C for 2 h under ultrasonication to remove the surface sizing agent and pollutants. After washing with ethanol and drying, unsized carbon fiber weaves were obtained. Subsequently, unsized carbon fiber weaves were immersed into 50 mL of a HNO_3_/H_2_SO_4_ (1/1, *v*/*v*) mixture at 60 °C for 15 min. Then, carbon fiber weaves were continually washed with deionized water until the residual solution was neutralized and then dried at 70 °C for 1 h. These are denoted as oxidized carbon fiber weaves.

### 2.3. Chemical Grafting of CNTs

Oxidized carbon fiber weaves were placed into melamine/tetrahydrofuran (THF) solution (1 × 10^−4^ mol/L). Subsequently, 10 mg of HATU were ultrasonically dissolved in the solution and reacted at 55 °C for 4 h. Then, carbon fiber weaves were removed and washed several times with THF and deionized water to remove unreacted melamine. The purpose of this step is to provide attachment points for CNTs on the carbon fibers surface. Next, carbon fiber weaves were added to CNT/N, N-dimethylformamide (DMF) solution (listed in Table 1), and 10 mg of HATU was separately added. The reaction was carried out at 90 °C for 4 h. After washing with DMF and deionized water, the carbon fiber weaves were dried in a blast drying oven.

### 2.4. Lap Joint Test Preparation

**Figure 1:**
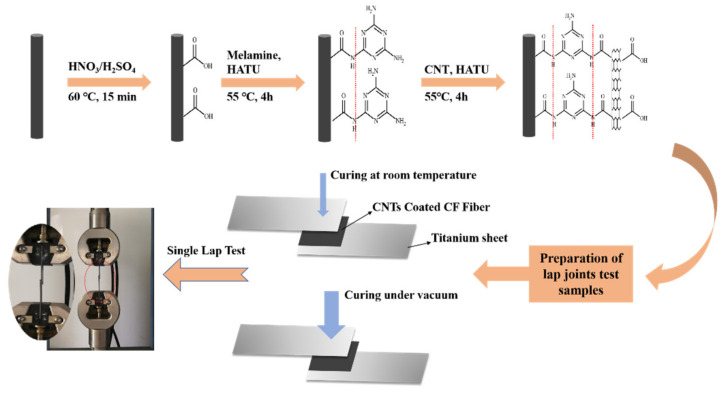
Schematic diagram of the experimental process.

First, titanium alloy sheets (100 × 25 × 1.5 mm) were subjected to physical sanding treatment and then washed with absolute ethanol to remove metal surface contaminants. In this test, a carbon fiber fabric woven in the 0°/90° direction was used as a reinforced composite material with a total thickness of 0.5 cm, which was laid flat on the joint of two titanium plates. The experiment weighed 6 g YT-CC302Q low-viscosity quick-drying epoxy resin and 2 g corresponding curing agent. A batch of samples were heated to 50 °C for 1 h and then at 70 °C for 3 h under atmospheric pressure. Another batch of samples were cured at 50 °C for 1 h and then at 70 °C for 3 h under vacuum compression. Each sample code was prepared for five lap joint samples. The thickness of the single lap sample joint is 3.6 cm. The flow chart of the experimental process is shown in Figure 1.

### 2.5. Characterization

X-ray photoelectron spectroscopy (XPS) (Shimadzu, Tokyo Japan KRATOS AXIS SUPRATM), using a monochromated Al Kα (hv = 1486.6 eV) at a base pressure of 2 × 10^−9^ mbar was used to study the elemental composition. The fixed transmission energy of the wide-scan energy analyzer was 160 eV and that of the narrow-scan energy analyzer was 20 to 40 eV. The surface morphologies of carbon fibers and the micro-morphology of the laminate after failure were observed using SEM (Regulus 8220, FEI, Hillsboro, OR, USA). The SEM was set to a back scattered electron image with 15 kV accelerating voltage, high vacuum, and high probe current. In this experiment, sample preparation and testing conditions were strictly carried out in accordance with the GB-T7124-2008 Adhesive Tensile Shear Strength Measurement Standard. The instrument used for the single-shear tensile shear mechanical property test is the CMT4254 microcomputer-controlled electronic universal testing machine produced by Shenzhen Xinsansi Material Testing Co., Ltd., Shenzhen, China. The test speed is 1 mm/min, and the test temperature is room temperature. The bonding strength was calculated according to the following equation:

(1) W=P/F=P/ab

where *P* is the maximum destructive load recorded, *F* is the lap area, ‘*a*’ is the long lap joint, and ‘*b*’ is the lap joint width. Each group was tested with no less than five pairs, and the lap area of the lap joint was calculated according to the actual measured value after each pair of test pieces was destroyed to an accuracy of 0.01 cm^2^.

## 3. Results and Discussion

### 3.1. Surface Chemistry Characteristics of Carbon Fiber

**Figure 2:**
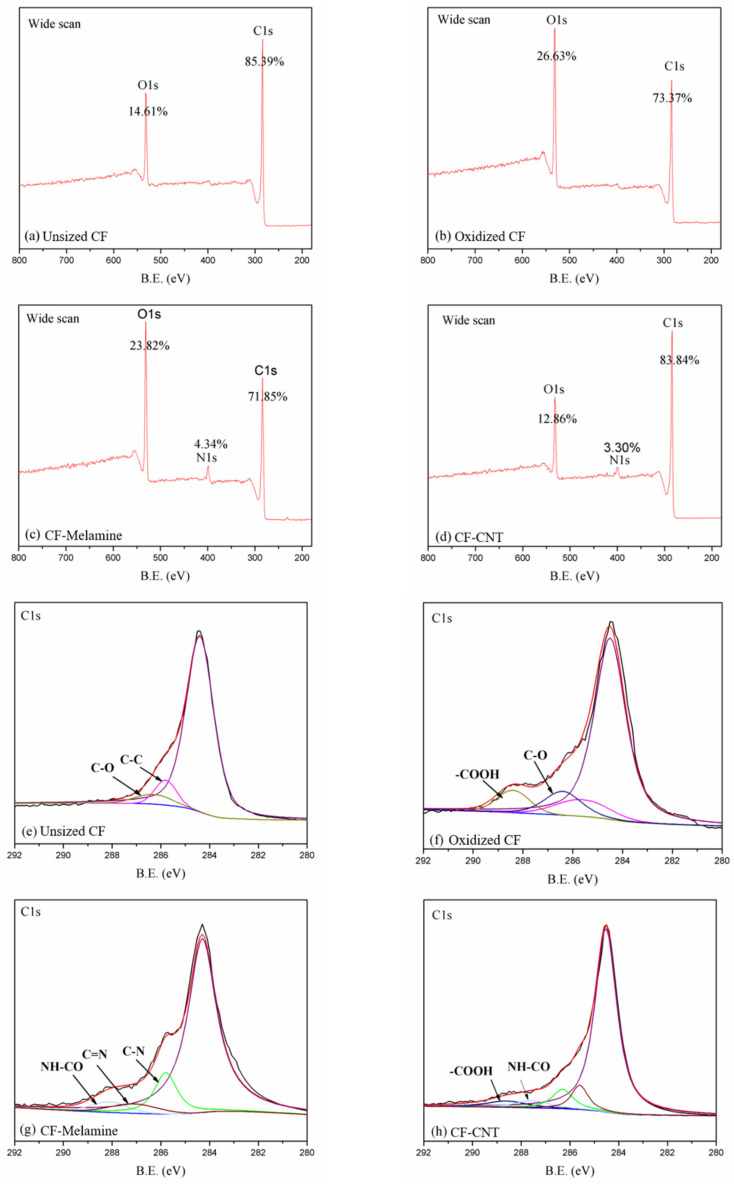
Wide-scan XPS spectra and C1s high-resolution XPS elemental spectra of (**a**,**e**) Unsized carbon fiber, (**b**,**f**) oxidized carbon fiber, (**c**,**g**) CF-Melamine, and (**d**,**h**) CF-CNT. (For interpretation of the references to color in this figure legend, the reader is referred to the web version of this article.)

The surface elements of each material generated at each step of the carbon fiber process were evaluated by XPS analysis. The wide-scans of carbon elements are shown in Figure 2a–d, and the change in the relative content of the carbon fiber surface is recorded in Table 2. From Table 2, the relative content of oxygen on the carbon fiber surface increased to 26.63% after mixed acid treatment. This indicates that the acid treatment increased the oxygen-containing functional groups on the surface of the carbon fibers, which increased the surface polarity of the carbon fibers. Furthermore, the presence of nitrogen (4.34%) demonstrates that melamine has been successfully grafted onto the surface of carbon fiber, and the subsequent significant increase in carbon resulted in successful CNT grafting. To more clearly demonstrate the state of the elements in each step of the process, Gaussian fitting for the high-resolution spectrum of C1s is highlighted in Figure 2e,f. According to Figure 2e, the highest peak at 284.4 eV is designated as Csp^2^ and Csp^3^ in the carbon fiber structure, and the peaks at 285.6 and 286.3 eV are assigned to the C-C and C-O bonds. The appearance of the C-O bond is caused by the purification process of the carbon fiber [32]. A new peak, the-COOH bond, appearing at 288.9 eV, is classified as the result of the mixed acid treatment (Figure 2f). In Figure 2g, the C-N (285.8 eV) and C=N (287.6 eV) bonds can be attributed to the melamine structure [31]. Moreover, the presence of NH-CO (288.0 eV) is a result of the reaction between-COOH and-NH_2_ and reveals that melamine is successfully grafted onto the surface of the carbon fiber. In Figure 2h, the newly occurring COOH peak at 288.9 eV can be assigned to the carboxyl-functionalized CNT, indicating the appearance of CNTs [33]. The change in XPS results confirms the successful grafting of CNTs on the carbon fiber surface.

### 3.2. Surface Morphology of Carbon Fiber

**Figure 3:**
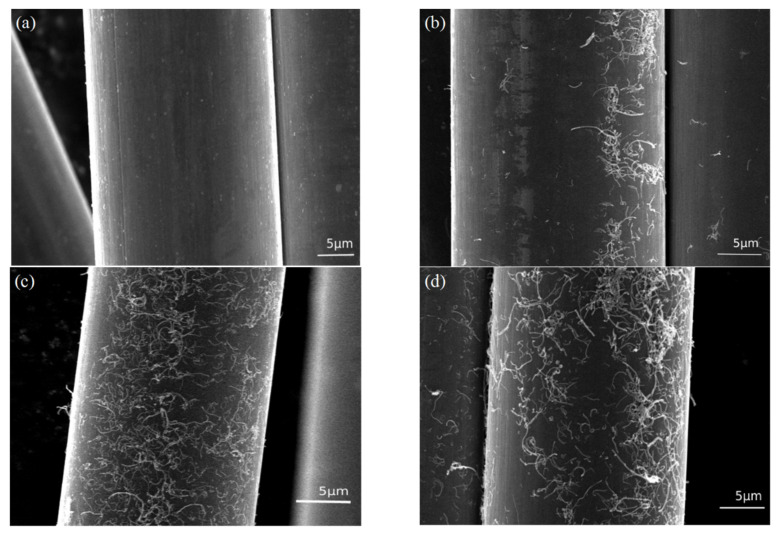
Scanning electron microscopy (SEM) images of the surface morphologies of carbon fiber: (**a**) Unsized carbon fiber, (**b**) CF-CNT_0.1_, (**c**) CF-CNT_0.2_, and (**d**) CF-CNT_0.3_.

**Figure 4:**
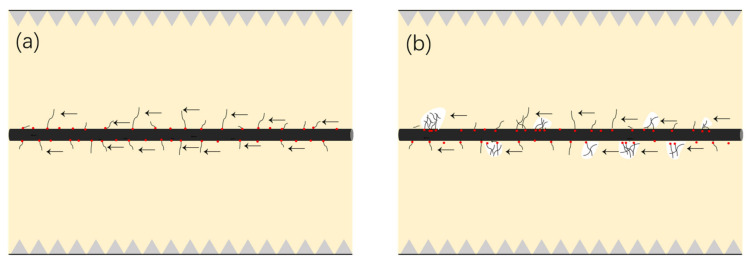
Schematic diagrams of carbon nanotube (CNT) enhancement: (**a**) optimal concentration of grafted CNTs; (**b**) a high concentration of CNTs. (The red dots indicate melamine, the arrows indicate the resistance to shearing forces, and the white parts indicate resistance to resin penetration.)

SEM images in Figure 3 show the surface microstructure of carbon fibers treated with different concentrations of CNT solution. Initially, the clean surface of the unsized carbon fiber is presented in Figure 3a. Without sizing, there are few active groups for any substances to bond, intentionally or unintentionally. Figure 3b–d depict the surfaces of the carbon fibers grafted from 0.1, 0.2, and 0.3 mg/mL CNT solutions, respectively. Compared to Figure 3a, it was observed that CNTs are grafted onto the carbon fiber surface. The presence of CNT increases the contact area of carbon fiber and resin and can be embedded into the epoxy resin, which causes the separation of carbon fiber and resin to require relatively high strength. When subjected to shear stress, CNTs can also hinder the propagation of cracks and better transfer the stress to the carbon fiber [34]. In addition, according to reports in the literature [35], unreacted melamine on the fiber surface can locally chemically bond with epoxy resin, enhancing the fracture toughness of the laminate (Figure 4a). Figure 3b shows that CNTs cannot completely cover the surface of the carbon fiber at a 0.1 mg/mL CNT solution. As the CNT solution concentration reaches 0.2 mg/mL, CNTs can be evenly distributed on the surface of carbon fiber (Figure 3c). When the CNT solution concentration continues to increase, CNTs will agglomerate on the surface (Figure 3d), which is not conducive to interfacial bonding between fibers and resins. Too many CNTs on the surface of CF will cause local stress concentration and reduce energy dissipation. Moreover, the narrow gaps between dense CNTs will limit the penetration of resin on the surface of the carbon fiber and lead to weak interfacial adhesion between the carbon fiber and the resin (Figure 4b). Thus, it is recommended that a 0.2 mg/mL CNT solution is the optimal concentration in this work.

### 3.3. Lap Joint Testing

**Figure 5:**
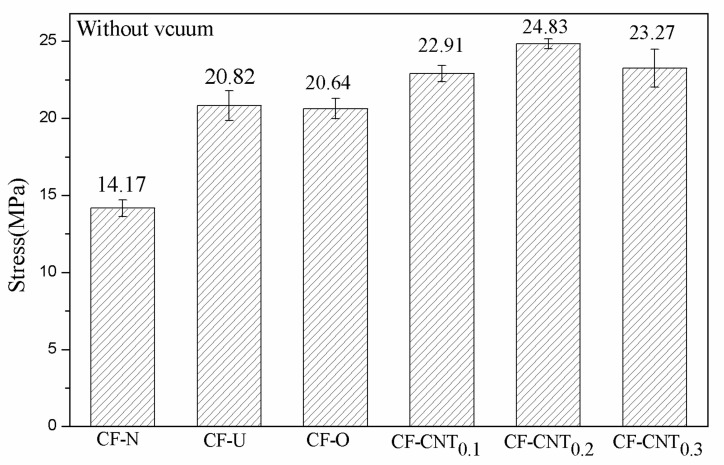

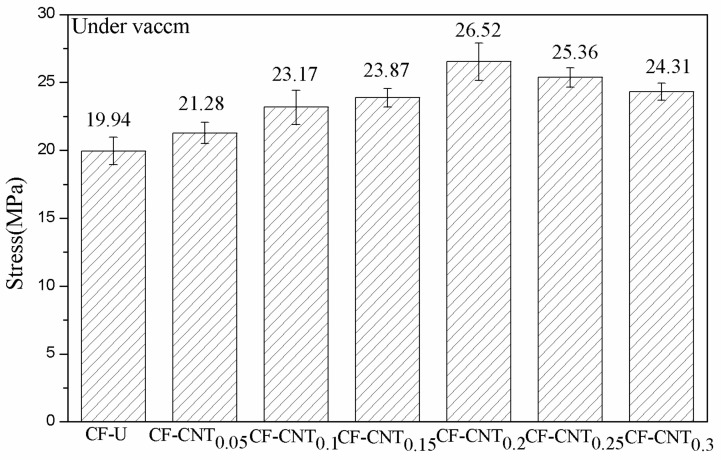
Bond strengths of single lap samples.

The bond strengths of the samples under different curing conditions are recorded in Table 3 and Table 4. The bond strength trend is shown graphically in Figure 5. In Table 3, CF-N is a control sample of two TA2 sheets bonded without carbon fiber, and its bond strength is 14.17 MPa. With the addition of unsized carbon fiber, the bonding strength of the CF-U increased by 46.93% to 20.82 MPa. After the carbon fibers were oxidized, the bond strength of CF-O showed little decrease. Excessive acid treatment can impair the performance of carbon fiber, but strict control of oxidation time does not have a significant impact. Additionally, liquid phase oxidation has milder properties than other oxidation methods [36]. When the carbon fibers are grafted under 0.1, 0.2, and 0.3 mg/mL CNT solutions, the bond strength increases by 61.68%, 75.23%, and 64.22%, respectively. The bond strength shows a maximum at a concentration of 0.2 mg/mL of CNT solution. At a concentration of 0.3 mg/mL CNT solution, the local agglomeration of CNTs on carbon fibers’ surface led to a decrease in the bond strength of the sample. These phenomena are also consistent with the results obtained from the surface topography observed under SEM. Table 4 indicates the bond strengths of the samples cured under vacuum compression. Compared to Table 3, all the sample bond strengths were improved except CF-U. Specifically, the bond strength of the sample grafted at a concentration of 0.2 mg/mL was most significantly improved and increased by 87.16% to 26.52 MPa. Under vacuum compression, the pressure causes the CNTs on the surface of the carbon fiber to become more embedded in the resin matrix, enabling the carbon fiber and epoxy resin to bond more closely. At atmospheric pressure, the lower pressure is a challenge for the resin to fully penetrate the surface of the carbon fiber grafted with CNTs [37]. In addition, since the surface of the unsized carbon fiber is smooth and there is no CNT grafting, the vacuum atmosphere has little effect on the bond strength of CF-U.

**Figure 6:**
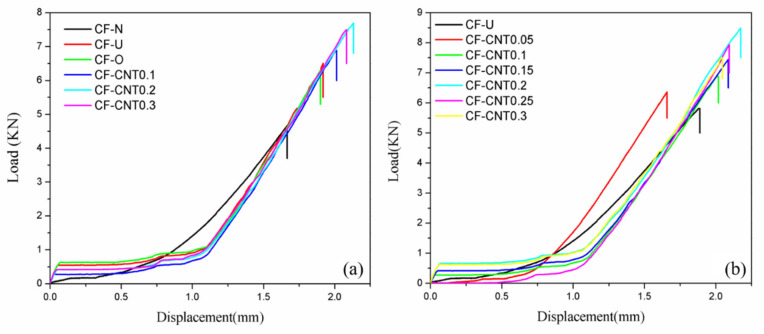
Relationship between load and displacement of the lap joint test: (**a**) samples cured at atmospheric pressure; (**b**) samples cured under vacuum.

Figure 6 shows the typical load–displacement curve of the samples. The lap joint bond failure of the samples is primarily divided into two processes. The first stage is the linear elastic deformation stage. During this phase, the change in displacement is small at the same time as the loads primarily shared by titanium alloy sheets and carbon fibers. As the loads continue to increase, the titanium alloy sheets reach a yield point and the curve enters the second stage, plastic deformation. At this stage, the loads are generally assumed by the bonding interface of the carbon fibers, epoxy resin, and titanium alloy sheets. Meanwhile, the displacement continuously increases with load increases. When the loads reach a maximum, fracture failure occurs at the interface of the samples, which is an irreversible process. At this point, the lap joint experiment is completed.

### 3.4. Analysis of Micro-Morphology

**Figure 7:**
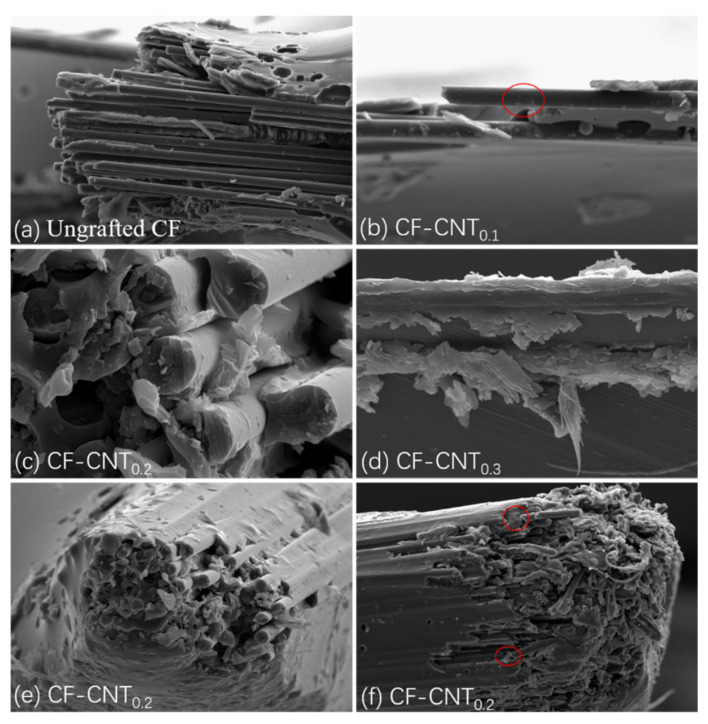
Microscopic morphology of laminate failure: (**a**) ungrafted CNT samples; (**b**) samples grafted with 0.1 mg/mL CNT concentration; (**c**,**e**,**f**) samples grafted with 0.2 mg/mL CNT concentration; (**d**) Samples grafted with 0.3 mg/mL CNT concentration.

Generally, there are three interfacial enhancement mechanisms for CNT-grafted carbon fibers, namely: (i) CNTs are detached from the carbon fiber surface; (ii) CNTs are pulled from the matrix; (iii) tensile failure of CNTs (fracture). When subjected to shear stress, the specific failure mode of CNTs depends on its interfacial bonding ability with fibers or resins, as well as its own mechanical properties. Figure 7 provides the microstructure of the laminate failure. It can be observed from Figure 7a that the fibers of ungrafted CNTs can be easily pulled out of the resin, and the surface is clean, with almost no residual resin. Figure 7b–d are fibers grafted with different concentrations of CNTs. It can be found that there are obvious resin residues on the fiber surface, which shows that the presence of CNTs significantly enhances the bonding strength between the fiber and resin. Since the grafting concentration in Figure 7b is too low, there is little resin remaining, and it is accompanied by the appearance of fiber pull-out. The carbon fiber surface in Figure 7c,d is covered with a large quantity of CNTs, so a great amount of resin remains on the surface. In addition, when the surface of the carbon fiber is rough after grafting carbon nanotubes, it cannot be easily pulled out of the resin, as shown in Figure 7e. When the force is too large, the fiber will be pulled out (Figure 7f). Fiber breakage requires energy absorption, which further improves the interfacial bonding properties. However, the phenomenon of pulling out and breaking of CNTs cannot be seen from the Figure 7 because most of the CNTs do not grow vertically on the fiber surface. It is completely adhered to the carbon fiber, which can be discovered from the surface morphology of the fiber in Figure 3. There is a large number of carboxyl groups on the surface of carboxylated CNTs, and each carboxyl position may react with the amino group on the surface of the carbon fiber grafted with melamine, resulting in the CNTs being unable to be vertically grafted on the fiber surface. The main role of CNTs in this experiment is to improve the roughness of the fiber surface and the contact area between the fiber and the resin, making the fiber difficult to be pulled out. The cohesive failure causes it to remain on the fiber surface, which consumes energy and enhances the overall bond strength of the laminate.

## 4. Conclusions

Chemical grafting of CNTs onto the surface of carbon fibers is effective for the interfacial property improvement of FMLs. XPS analysis shows that the surface of carbon fiber treated with mixed acid produces a large number of oxygen-containing functional groups, and the relative oxygen content increases from 14.61% to 26.63%. In addition, the appearance of nitrogen indicates successful melamine grafting. In observing the surface morphology of carbon fibers, CNTs can be evenly distributed on the surface of carbon fibers at a concentration of 0.2 mg/mL CNT solution, causing a bond strength increase of 87.16% to 26.52 MPa. With CNT solution concentration increased to 0.3 mg/mL, CNTs locally agglomerate on the surface of carbon fibers, and the bond strength is significantly reduced. The emergence of CNTs effectively improves the mechanical interlocking between carbon fibers and epoxy resin, enhances the interfacial bonding performance of FMLs, and generates a change in interfacial failure mode from adhesive to cohesive failure. In summary, the performance of FMLs can be greatly improved with fiber modification.

## Figures and Tables

**Table 1:** Cured samples prepared from variously treated carbon fiber weaves.

Sample Code	Variously Treated Carbon Fiber Weaves
CF-N	No carbon fiber weaves added
CF-D	Unsized carbon fiber weaves
CF-O	Oxidized carbon fiber weaves
CF-CNT_0.05_	Grafted carbon fiber weaves with CNT concentration of 0.05 mg/mL
CF-CNT_0.1_	Grafted carbon fiber weaves with CNT concentration of 0.1 mg/mL
CF-CNT_0.15_	Grafted carbon fiber weaves with CNT concentration of 0.15 mg/mL
CF-CNT_0.2_	Grafted carbon fiber weaves with CNT concentration of 0.2 mg/mL
CF-CNT_0.25_	Grafted carbon fiber weaves with CNT concentration of 0.25 mg/mL
CF-CNT_0.3_	Grafted carbon fiber weaves with CNT concentration of 0.3 mg/mL

**Table 2:** The relative content of surface elements of different treated carbon fibers.

Sample	C	O	N
unsized CF	85.39%	14.61%	0
oxidized CF	73.37%	26.63%	0
CF-Melamine	71.85%	23.82%	4.34%
CF- CNT	83.84%	12.86%	3.30%

**Table 3:** Bond strength of samples cured under atmospheric pressure.

Sample Code	Stress (MPa)	SD	Increase (%)
CF-N	14.17	0.54	-
CF-U	20.82	0.96	46.93
CF-O	20.64	0.65	45.66
CF-CNT_0.1_	22.91	0.53	61.68
CF-CNT_0.2_	24.83	0.32	75.23
CF-CNT_0.3_	23.27	1.23	64.22

**Table 4:** Bond strength of samples cured under vacuum compression.

Sample Code	Stress (MPa)	SD	Increase (%)
CF-U	19.94	1.01	40.72
CF-CNT_0.05_	21.28	0.78	50.18
CF-CNT_0.1_	23.17	1.26	63.51
CF-CNT_0.15_	23.87	0.69	68.45
CF-CNT_0.2_	26.52	1.38	87.16
CF-CNT_0.25_	25.36	0.72	78.96
CF-CNT_0.3_	24.31	0.63	71.56
